# Teleassessments for Enrollment of Adults With Physical or Mobility Disability in a Home-Based Exercise Trial in Response to COVID-19: Usability Study

**DOI:** 10.2196/29799

**Published:** 2021-11-18

**Authors:** Jereme Wilroy, Byron Lai, Madison Currie, Hui-Ju Young, Mohanraj Thirumalai, Tapan Mehta, John Giannone, James Rimmer

**Affiliations:** 1 University of Alabama at Birmingham Birmingham, AL United States

**Keywords:** telehealth, disability, COVID-19, exercise, assessments, feasibility, mHealth, teleassessment, mobility impairment, home exercise, participation, physical disabilities

## Abstract

**Background:**

The Scale-Up Project Evaluating Responsiveness to Home Exercise And Lifestyle Tele-Health (SUPER-HEALTH) initiative is a large randomized controlled study that aims to overcome logistical barriers to exercise via telehealth for people with physical disabilities. However, at the start of the COVID-19 pandemic, enrollment was halted due to limited operations at the testing site, which included no onsite visits that involved participant data collection. In response to the limited operations, a modified data collection protocol was developed for virtual enrollment of study participants.

**Objective:**

This paper presents feasibility data on using teleassessments to enroll people with mobility impairment into a home-based exercise trial.

**Methods:**

The modified protocol replaced onsite enrollment and data collection visits with teleassessments using a computer tablet and testing equipment that was shipped to the participants’ home address prior to the synchronous teleassessments conducted by an exercise physiologist through Zoom. The participants were mailed a teleassessment toolkit that included a digital blood pressure cuff, spirometer, hand dynamometer, mini disc cone, and measuring tape (to complete standardized testing). The teleassessment measures included resting blood pressure and heart rate, forced vital capacity, grip strength, Five Times Sit to Stand, and Timed Up and Go. Feasibility metrics included technological effectiveness, efficiency, and safety. The technological effectiveness of the telehealth assessment was determined by the percentage of sessions completed without technical issues with ≥90% criteria set a priori. Efficiency was measured by a session duration of ≤2 hours. Safety was measured by the number of adverse events related to the teleassessments reported.

**Results:**

Data from 36 participants were included in this feasibility study, and 34 (94%) participants completed all teleassessments without technical issues. For efficiency, the teleassessment sessions were completed in a mean time of 65 minutes and a maximum session length of 110 minutes. There were no adverse events reported to indicate concerns with the safety of teleassessments.

**Conclusions:**

The modified teleassessment protocol, in response to COVID-19 restrictions, may be a feasible process for enrolling adults with mobility impairment into a home exercise trial who otherwise would have not been able to participate.

**Trial Registration:**

ClinicalTrials.gov NCT03024320; https://clinicaltrials.gov/ct2/show/NCT03024320

## Introduction

People with physical disabilities are at greater risk for primary and secondary health conditions compared with people without a disability [1-3]. These include primary health conditions, such as heart disease and diabetes, and secondary conditions, including pain, depression, sleep disturbance, spasticity, and many others [4,5]. Despite physical activity becoming an imperative public health priority for all demographics, there are still many existing barriers to physical activity. A few of these barriers include accessibility of facilities, opportunity for activity, and aesthetic or environmental attributes [6]. In order to overcome these barriers, people with physical disabilities must be given more options for home-based activity, thus eliminating many well-known community-based barriers [7,8].

The SUPER-HEALTH project, which stands for Scale-Up Project Evaluating Responsiveness to Home Exercise And Lifestyle Tele-Health, is an ongoing 48-week exercise trial assessing the utilization of a movement-to-music (M2M) intervention remotely delivered to various disability groups [8]. SUPER-HEALTH recruits individuals with a mobility impairment, defined as the inability to walk or difficulty walking as a means to exercise, and includes people with spinal cord injury (SCI), spina bifida (SB), multiple sclerosis (MS), stroke, limb loss, and other disabilities and chronic health conditions. SUPER-HEALTH was designed to deliver an exercise training system in the convenience of the home in order to avoid barriers associated with transportation for onsite exercising at a facility. However, project enrollment was halted by the University of Alabama at Birmingham due to COVID-19 restrictions involving onsite data collection. To overcome this issue, the project team developed teleassessment protocols that enabled the trial to continue enrolling eligible participants across the southeastern United States [9]. The purpose of this paper is to describe the feasibility of using teleassessments for enrolling people with physical disabilities into a home-based exercise trial by evaluating three feasibility components, namely technological effectiveness (ie, successful implementation without technical error), efficiency, and safety.

## Methods

### Description of SUPER-HEALTH

SUPER-HEALTH is testing a 48-week, home-based exercise training system among a large sample (N=648) of people with mobility impairments by comparing 3 groups: (1) attention control group (AC) receiving health promotion articles; (2) exercise group (M2M) receiving exercise videos and health promotion articles; and (3) exercise group receiving exercise videos, health promotion articles, and social networking features (M2M^plus^). Participants in each group received a Samsung Tab A 10.1 tablet, which was inserted into a protective case and customized with features pertaining to the study. These features included downloads of the study app [7], the Fitbit app with the Fitbit Charge 4 synced and connected by Bluetooth, and the Zoom app for communication. An email address was provided to the participant along with a Gmail widget configured on the tablet to communicate with research staff. Additionally, the exercise groups also received wrist weights.

This protocol was approved by the university’s Institutional Review Board (IRB) and is registered with ClinicialTrials.gov (NCT03024320) as a phase III clinical trial. We certify that all applicable institutional and governmental regulations concerning the ethical use of human volunteers were followed during the course of this research.

### Participants

Participants who were part of the SUPER-HEALTH study and who completed teleassessment were analyzed as part of this feasibility study. Textbox 1 provides lists of inclusion and exclusion criteria, which are the same as the main trial with the additional criteria concerning the completion of teleassessments. A convenience sample size of 36 was chosen to satisfy technological usability [10] and feasibility study recommendations [11]. In order to recruit participants for the study, a list of patient names and addresses from a health informatics database was obtained and study brochures were mailed out. The participants enrolled by calling, providing contact information through the study website, or contacting the study email.

**Inclusion and exclusion criteria.**

**Inclusion criteria**

- Self-report of a physical disability or mobility impairment
- 18 to 74 years of age
- Not currently enrolled in a structured exercise program over the past 6 months
- Have the ability to use upper, lower, or both sets of extremities to exercise
- Have the ability to converse and read English
- Agree to receiving emails to complete consent, surveys, and teleassessments

**Exclusion criteria**

- Medically unstable to perform home exercise as determined by their physician
- Cognitive impairment that may preclude self-directed daily activities
- No wireless internet in home (can include hotspot with unlimited data)

### COVID-19 Modifications

In order to avoid the need for participants to commute to the laboratory for onsite data collection, our team developed protocols for synchronous remote assessments (teleassessments) and virtual orientation. The new teleassessment protocol was used for the original study from this point forward. In the following section, we will discuss some aspects of the modified protocol.

#### Consent and Survey Completion

As with the original study protocol, consent and survey data collection involved emailing links to participants, connecting them to an electronic form via REDCap (electronic data capture system). Once the participants signed and submitted the electronic consent, they would automatically receive surveys from REDCap. The questionnaires included a demographic and health history questionnaire; a medication list; Godin Leisure Time Exercise Questionnaire (GLTEQ) [12]; social cognitive theory questionnaires for exercise self-efficacy [13], barriers to physical activity [14], outcome expectations [15], and social support [16]; a set of Patient Reported Outcomes Measurement Information System (PROMIS) short forms [17] to assess quality of life; and an eHealth Literacy Scale [18]. After all of the surveys were completed, the research staff packed and shipped the assessment and training equipment to the participants’ provided home address, as part of the modified protocol.

#### Teleassessment Package

**Figure 1:**
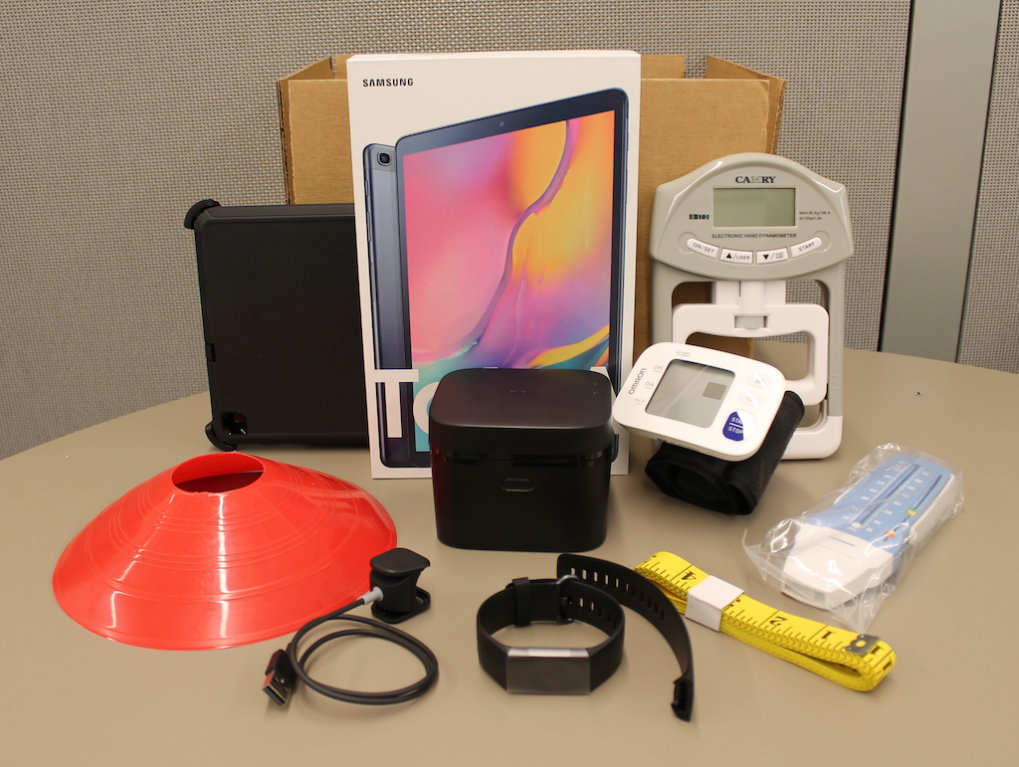
Teleassessment equipment, which included a digital blood pressure cuff, a hand dynamometer, a spirometer, a mini disc cone, and a 3-meter measuring tape.

Teleassessment equipment mailed to each participant included a digital blood pressure and heart rate monitor (US $45), hand-held dynamometer (US $30), spirometer (US $25), mini disc cone (US $1), and soft measuring tape (US $2). All of the equipment was lightweight and ready to use out of the box due to preparation by a research assistant prior to shipping. Teleassessment equipment was packaged along with the Samsung Tab A tablet (US $200) and Fitbit Charge 4 device (US $120) (Figure 1). Each participant was informed of equipment shipment date and expected delivery date, and the package was tracked by the research assistant. A brief instruction sheet was included and simply stated to keep the tablet charged, await call from tester to schedule virtual testing appointment, and email that equipment has been received, along with the option to contact study staff with any questions. Once the participant received their equipment, the teleassessor reached out to schedule the participant for their baseline teleassessment session. The participants kept all equipment for the duration of the study and beyond.

#### Teleassessment Session

**Figure 2:**
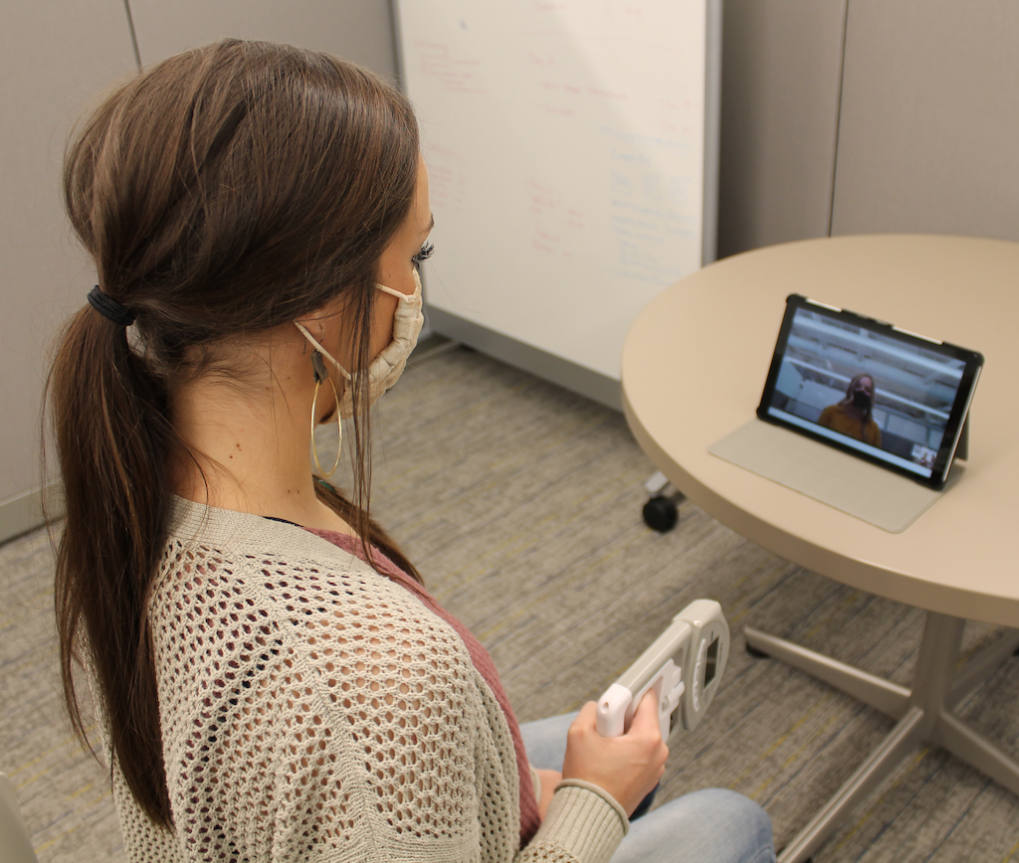
An example of a person completing grip strength assessment with teleassessor on videoconference.

A single teleassessor, an exercise and sport science lab assistant with a master’s in exercise physiology, was used to complete the teleassessment session. At the start of the session, the teleassessor instructed the participant to make sure the tablet was on a table propped on case stand and ensured the participant was in full view. They would then go through a script concerning a plan for adverse events (eg, fall) and internet connectivity issues. The session included the following assessments: anthropometrics (2 resting blood pressure readings, 2 resting heart rate readings), grip strength, peak expiratory volume, Five Times Sit to Stand, and Timed Up and Go (Figure 2). All measures were completed based on the participants’ disability and level of function. For example, if someone was unable to walk, they would not complete the Five Times Sit to Stand and Timed Up and Go tests. For exercise groups, the participant’s functional group was assigned to ensure they received the appropriate exercise videos, which was (1) able to stand, (2) hemiparesis, or (3) sitting only. This assignment was based on the participant’s diagnosis, 14-second cut-point of Timed Up and Go (ie, assigned to seated exercise group if greater than 14 seconds), and participant preference.

#### Virtual Equipment Orientation

Once the teleassessment was completed, the participant received a virtual equipment orientation via Zoom to notify which group they had been allocated to. They were also mailed a US $25 Visa gift card via UPS mail delivery. The purpose of this meeting was to inform the participants of which group they had been randomized to and how to use each piece of equipment correctly (ie, Fitbit and tablet) for the purposes of the intervention.

### Data Collection for Feasibility

Since the COVID-19 modifications were focused on the application of a technological medium (videoconferencing), we chose to assess the feasibility of the teleassessment process [19] through 3 metrics of technological usability: technological effectiveness, efficiency, and safety [10].

#### Technological Effectiveness

Effectiveness was defined as the percentage of sessions completed successfully without technical or logistical difficulty. These difficulties could have included any issues with information communication technology (ie, tablet, internet, videoconference software, or assessment equipment) experienced by the participant or teleassessor. Physiologic testing issues, such as high resting blood pressure or heart rate or an inability to complete an assessment due to mobility level (eg, wheelchair use), were excluded because these cases would have also excluded the participants from completing assessments onsite. We established an a priori criterion for acceptable effectiveness at 90% used previously with this population [20]. A high criterion of 90% was chosen because we wanted the participants to have minimal issues and a high-quality experience during the teleassessments.

#### Efficiency

Efficiency was defined as the amount of time to complete teleassessments. The timer started once the participant and teleassessor logged on to the videoconferencing session and ended at the point of logging off. The criterion for reasonable efficiency was set at 2 hours or less a priori as this was the time allotted for onsite visits.

#### Safety

Adverse events, defined by the university’s IRB as “…any untoward or unfavorable medical occurrence in a human subject,” were reported by the teleassessor with a written description of the event. The event was then recorded in a medical oversight form, which was reviewed by the study physician who classified the event as serious or non-serious and whether it was due to study procedures. Any adverse event was then reported to the IRB. Examples of adverse events would include falls or injury during testing.

## Results

### Demographics

Data from a convenience sample of 36 people were included within this feasibility report. The participants were primarily female and African American (Table 1). These participants were located in southeastern United States.

**Table 1:** Demographic data of participants (N=36).

Characteristic		Value
Age (years), mean (SD)		51.08 (16.64)
**Gender, n (%)**		
	Male	11 (31)
	Female	25 (69)
**Race, n (%)**		
	African American	21 (58)
	White	13 (36)
	Other	2 (6)
**Disability, n (%)**		
	Spinal cord injury	5 (14)
	Spina bifida	5 (14)
	Arthritis	11 (31)
	Stroke	3 (8)
	Other	12 (33)
**Device, n (%)**		
	Wheelchair only	6 (17)
	Cane only	6 (17)
	Motorized scooter only	2 (6)
	Other	1 (3)
	No assistive devices	11 (31)

### Feasibility

#### Technological Effectiveness

Out of the 36 participants in the sample, 34 (94%) completed all teleassessments without issues, which met the criteria for acceptability (>90%) set a priori. This resulted in the successful completion of 188 assessments. The reasons for the 2 participants not completing all teleassessments included: incomplete spirometry test due to confusion regarding manual peak flow meter and incomplete Timed Up and Go assessment due to technical difficulties with videoconferencing on the tablet.

#### Efficiency

The mean time for the completion of a teleassessment session for the sample of 36 participants was 65.03 (SD 15.51) minutes, which met our a priori criterion for acceptability (maximum session length: 110.00 minutes). Therefore, the criterion for completing the sessions in under 2 hours was met by all participants (N=36, 100%) of the sample. The reasons reported for the longest session times included tablet not being charged prior to the visit and internet connection issues.

#### Safety

There were no adverse events reported from the sample of 36 teleassessment sessions.

## Discussion

### Principal Considerations

Many trials have been halted due to the restriction of onsite visits by research participants during the COVID-19 pandemic. Given that a significant percentage of the population is either not yet vaccinated or elects not to be vaccinated [21], exercise testing may need to continue to be remotely conducted in the foreseeable future. In addition, other rehabilitation researchers have endorsed the use of functional assessments not requiring costly or complex equipment or specialized training [22]**.** There is a strong need for researchers to have effective, safe teleassessment protocols that are equivalent to in-person testing.

SUPER-HEALTH is inclusive to varying levels of disability and functionality of all participating users. Thus, the use of telecommunication, teleintervention, and interactive methods were specifically designed to collect the same data virtually as it is carried out onsite. Along with these modifications, the virtual face-to-face interactions between the participants and the study staff suggest that telecommunication is still personable and equally as effective [23]. Prior to COVID-19, most of the enrollment process was completed remotely: phone screen, obtaining medical clearance, electronic consent, and electronic surveys. However, the physiological assessments as well as the equipment orientation were still conducted onsite, which often led to several missed visits and individuals lost to contact. The newly modified protocol utilizing telehealth to conduct these assessments and orientation has enabled the enrollment process to be completed remotely. This eliminated the need for transportation, which is the primary barrier to accessing community exercise programs among patients with physical disability [6]. The teleassessment package was affordable at around US $100. In addition, the teleassessment session duration was almost half that of the onsite, which, along with fewer missed sessions, saved cost for lab assistant’s time. Lastly, the cost for using lab space was substantially more than the smaller tele-suite the lab assistant was able to use for the teleassessment sessions.

These findings are an extension of our work related to modifying specific fitness assessment protocols for people with disabilities to be completed remotely [9]. One thing to note is that elements of the teleassessments, such as laptop camera angle and variability of testing environment and setups, might affect the validity and reliability of the tests. Future research on teleassessments should focus on modifying more testing protocols to ensure the reliability and validity of the data collected. Additionally, future studies should compare data collected onsite with data collected through teleassessments for any statistically significant differences. A preliminary analysis of the standard deviations between teleassessments and onsite assessments with the current sample did not indicate significant differences; however, a higher-powered validity comparison study and a test-retest study should be completed.

### Implications

A challenge often found in exercise trials involving people with disabilities is recruitment. One of the most limiting factors is enrolling large samples in exercise research for this population. A scoping review reported a lack of descriptive details on study participants and noted that many of the published studies have small sample sizes, which were primarily due to individuals being excluded, declining to participate, or dropping out [24]. Barriers reported by patients with physical disability to enrolling in an exercise trial include transportation, scheduling conflicts, secondary health conditions, and difficulty starting a program [25]. The utilization of teleassessments allows researchers to circumnavigate these reported barriers by increasing their ability to enroll more participants. For example, prior to modifying the assessment procedures to enable remote assessment for SUPER-HEALTH, a primary barrier reported by participants was transportation to the research lab. However, the teleassessment protocols have more than doubled the rate of recruitment, and participants are now able to enroll from the convenience of their home. Although 1 onsite laboratory test (ie, submaximal cardiorespiratory endurance) could not be performed remotely and was excluded from the teleassessment protocol, all of the other functional and self-reported measures were successfully conducted remotely. Our current battery of tests provides a starting place for remotely collecting data from the participants. While many studies had to temporarily shut down due to the COVID-19 pandemic, we were able to continue our research by implementing a teleassessment protocol that was found to be effective, efficient, and safe. Future research involving hard-to-reach populations (ie, people with disabilities) should continue to explore the use of teleassessment and tele-exercise in order to reach a broader, more generalizable segment of the population [22].

### Limitations

It is important to recognize the limitations of the study. First, the study excluded people who did not have internet access at home, which may have affected the representativeness of the sample. Second, the study included 12 participants who were non-ambulatory (wheelchair users) and were unable to complete two of the functional tests (Five Times Sit to Stand and Timed Up and Go), which reduced the time for completing the teleassessment session. Third, although the sample size meets the recommendations for feasibility studies [10,11], it may not be fully representative of the final sample size of the larger trial, which will be determined at the end of the trial to inform whether data can be merged. Fourth, since the assessments were delivered through telehealth, it is important to note the possible limitations in generalizability due to an inability to account for contextual factors varying among the participants’ location of testing [23]. Finally, the results should be interpreted with caution. This is because the study outcomes were chosen based upon arbitrary criteria, and there are no established criterion for the study outcomes that are considered to be feasible or acceptable.

### Conclusions

The use of teleassessments to enroll patients with physical disability into an exercise trial may be technologically effective, efficient, and safe. Due to the COVID-19 pandemic, many clinical trials have been suspended. The procedures described in this study can be replicated by researchers and health professionals to circumvent the barriers to conducting exercise trials during the pandemic.

## Abbreviations

IRB: Institutional Review Board
M2M: movement-to-music
SUPER-HEALTH: Scale-Up Project Evaluating Responsiveness to Home Exercise And Lifestyle Tele-Health
